# Development and Integration of a Digital Twin Model for a Real Hydroelectric Power Plant

**DOI:** 10.3390/s24134174

**Published:** 2024-06-27

**Authors:** Mustafa Ersan, Erdal Irmak

**Affiliations:** 1 Department of Electrical and Electronics Engineering, Graduate School of Natural and Applied Sciences, Gazi University, Ankara 06560, Türkiye; mustafa.ersan@contectus.com; 2 Department of Electrical and Electronics Engineering, Faculty of Technology, Gazi University, Ankara 06560, Türkiye

**Keywords:** digital twin, hydroelectric power plant, anomaly detection

## Abstract

In this study, a digital twin model of a hydroelectric power plant has been created. Models of the entire power plant have been created and malfunction situations of a sensor located after the inlet valve of the plant have been analyzed using a programmable logic controller (PLC). As a feature of the digital twin (DT), the error prediction and prevention function has been studied specifically for the pressure sensor. The accuracy and reliability of the data obtained from the sensor are compared with the data obtained from the DT model. The comparison results are evaluated and erroneous data are identified. In this way, it is determined whether the malfunction occurring in the system is a real malfunction or a malfunction caused by measurement or connection errors. In the case of sensor failure or measurement-related malfunction, this situation is determined through the digital twin-based control mechanism. In the case of actual failure, the system is stopped, but in the case of measurement or connection errors, since the data are calculated by the DT model, the value in the specified region is known and thus there is no need to stop the system. This prevents production loss in the hydroelectric power plant by ensuring the continuity of the system in case of errors.

## 1. Introduction

Digital twins (DTs) are virtual representations or replicas of a physical object, system, or process. They are created using real-time data from sensors installed on the physical object or system. This digital construct has the ability to simulate, analyze, and predict the behavior and composition of the real entity [1]. DTs are employed across various industries, including manufacturing, healthcare, automotive, aerospace, and more. They enable organizations to effectively monitor and manage their systems, optimize operations, predict maintenance needs, and simulate scenarios in advance for better decision-making [2,3]. While DTs and industrial control systems (ICSs) share similarities in monitoring and managing industrial processes, they serve distinct purposes and employ different technologies. ICSs, utilizing PLCs, SCADA systems, and distributed control systems, focus on real-time control and automation to ensure immediate operational responsiveness within predefined parameters. In contrast, DTs leverage comprehensive data integration and advanced modeling techniques to provide a holistic view, enabling predictive analytics and strategic planning beyond immediate operational needs.

DTs have become indispensable across industries, revolutionizing how organizations manage assets, optimize processes, and make decisions [4,5,6]. In manufacturing, they simulate production lines, predict maintenance needs, and enhance operational efficiency [7,8,9]. In healthcare, DTs of patients enable personalized treatment plans and predictive health monitoring [3,10,11]. Urban planners use DTs to manage infrastructure, traffic flow, and urban development [12,13]. The aerospace and defense industries utilize DTs to simulate aircraft performance and mission planning [14,15]. Energy and utilities leverage DTs to optimize energy use and predict equipment failures [16,17,18]. Automotive industries use DTs for vehicle design, performance analysis, and predictive maintenance [19,20,21]. In the retail sector, DTs optimize store layouts, analyze customer behavior, and predict demand [22,23]. Telecommunications companies employ DTs to monitor network performance and plan upgrades [24,25]. Additionally, environmental monitoring relies on DTs to simulate and evaluate environmental conditions and predict natural disasters [26,27,28]. Across these diverse applications, DTs continue to drive innovation and efficiency, reshaping industries for the better.

In the energy sector, DTs play a crucial role in optimizing energy resources [29]. DTs of power plants, renewable energy facilities, and utility networks provide real-time monitoring and analysis capabilities, enabling operators to enhance efficiency and reliability while reducing costs and environmental impacts [30]. These digital systems simulate the behavior of physical assets, facilitating predictive maintenance to prevent costly downtime and optimize performance. Furthermore, DTs assist in grid management by predicting demand fluctuations, optimizing the energy distribution, and seamlessly integrating renewable energy sources into existing infrastructure [31]. By leveraging data analytics and simulation capabilities, DTs in the energy sector support the transition to a more sustainable and resilient energy system, integrating smart grids and fostering the adoption of new technologies such as energy storage and electric vehicles [32].

In hydroelectric power generation, DTs offer advanced tools to monitor, analyze, and optimize the performance of hydroelectric facilities. These twins model the entire hydropower system, including the turbines, generators, reservoirs, and related infrastructure. By integrating real-time data from sensors throughout the facility, DTs provide operators with comprehensive information about the operational status, efficiency, and health of critical components [33]. One primary application in hydropower is predictive maintenance [34]. Constantly monitoring equipment parameters such as the vibration, temperature, and pressure, DTs can detect early signs of potential malfunctions or performance degradation. This proactive approach allows maintenance teams to plan interventions during scheduled outages, minimizing unplanned downtime and reducing maintenance costs. Furthermore, DTs facilitate performance optimization by simulating various operational scenarios and analyzing the impact of different operating parameters on energy production and efficiency. Operators can use these simulations to fine-tune control strategies, adjust work schedules, and maximize energy output while adhering to regulatory constraints and environmental considerations [32]. Additionally, DTs support asset management by providing a comprehensive overview of the entire hydropower infrastructure. Operators can visualize asset health, track historical performance trends, and make data-driven decisions regarding asset life-cycle management, upgrades, and investments. Through these capabilities, DTs significantly contribute to the overall efficiency, reliability, and sustainability of hydroelectric power generation.

Although numerous studies on digital twin technology are presented in the recent literature, some of the most notable ones are categorized in Table 1, Table 2, Table 3 and Table 4. These tables provide an overview of the software, systems, studies, and structures employed in the development and application of DTs within the energy sector. Table 1 enumerates various software tools used, such as MATLAB, Ansys, and COMSOL, along with their corresponding references, highlighting their specific applications in creating DTs. Table 2 categorizes the systems where DTs are implemented, including power plants, wind turbines, and steam turbines, with references to relevant studies. Table 3 outlines different research studies on energy digital twins (EDTs) [18], detailing their primary purposes, such as virtual testing for design, process optimization, and fault detection, along with associated references. Table 4 describes the structural aspects of EDTs, emphasizing AI-based control and optimization, data-driven cooling tower analysis, machine learning with IoT support, and online power grid analysis, each supported by specific references. Collectively, these tables offer a comprehensive snapshot of the methodologies, applications, and research objectives in the domain of DTs for energy systems.

Anomaly detection plays a crucial role in sensor technology where misleading signals can lead to significant errors. Deep-learning techniques, particularly autoencoder-based neural networks, have emerged as effective tools for addressing this challenge. A realized study delves into the application of deep learning for anomaly detection in sensor signals, with a focus on semi-supervised-learning approaches using autoencoder networks. Studies in this area have shown that deep-learning models based on autoencoders are capable of accurately identifying anomalies in sensor data. Both vanilla autoencoder models and their integrated variants have demonstrated comparable levels of accuracy, ranging from 40% to 80% in detecting anomalies. However, it is worth noting that LSTM-based autoencoder models exhibited lower accuracy rates in these anomaly detection tasks, highlighting the need for further exploration and optimization of the model architectures for specific sensor signal anomaly detection scenarios [52].

Sensor anomaly detection is particularly critical for assessing water quality in remote areas where continuous monitoring is essential but challenging. In a realized research study, self-organizing feature maps (SFOMs) and hierarchical clustering (HC) have been effectively utilized for anomaly detection in water quality assessment. These methods have demonstrated successful impacts on the performance of monitoring systems, showcasing their ability to detect anomalies and improve the overall assessment accuracy. By employing SFOM-based techniques and HC, researchers have not only enhanced the performance but also reduced the number of sensors required, leading to cost-effectiveness in monitoring systems. This successful application of SFOMs and HC in water quality assessment signifies their potential in improving monitoring infrastructure and ensuring reliable data collection in remote and critical environmental settings [53].

A realized study evaluates the performance of an unsupervised anomaly detection algorithm specifically designed for car sensors using neural networks. This method prioritizes creating small anomaly detectors with reduced parameters and computational requirements to ensure efficient computation, especially in real-time applications. By focusing on predictive maintenance in the automotive industry, the algorithm aims to anticipate and manage failures proactively, leading to several benefits, such as improved design, financial optimization, and timely issue resolution. The study explores the use of recurrent and convolutional neural networks for analyzing car sensor time-series data and achieving effective anomaly detection without the need for labeled training data. Notably, smaller predictors derived from these neural network architectures demonstrate similar anomaly detection performance while reducing the overall complexity of the detection system. The best results obtained from the evaluation showcase 86 successful anomaly detections using a window size of 60 s, highlighting the algorithm’s potential for practical implementation in the automotive sector [54].

An explainable anomaly detection system (XADS) is designed to cater specifically to IoT sensors in monitoring systems that deal with categorical data for anomaly detection. It stands out by providing detailed explanations for anomalies detected in the sensor data, making it an excellent choice for users who require transparency and understanding of the detected anomalies. An XADS boasts high accuracy in anomaly detection while requiring limited parameter setting, making it efficient and practical for implementation. Additionally, its user-friendly interface enhances the monitoring experience, allowing users to navigate through detailed anomaly information easily and make informed decisions based on the explanations provided. Overall, an XADS serves as an explainable anomaly detection solution tailored for categorical sensor data, offering a blend of accuracy, simplicity, and user accessibility [55].

In the realm of IoT sensor platforms for anomaly detection in industrial equipment, deep-learning methods such as LSTM and autoencoders have garnered significant attention. These methods are utilized for their effectiveness in detecting anomalies in sensor data. A notable aspect is the comparison of autoencoders with other deep-learning architectures, like DNNs, LSTMs, and CNNs, on benchmark datasets. Autoencoders, in particular, exhibit promise for anomaly detection in IoT sensor data, showcasing their ability to detect anomalies across various operating conditions without requiring extensive feature engineering. This approach leverages a single model to efficiently detect anomalies, offering a streamlined solution for minimal configuration and effective anomaly detection in diverse industrial settings [56].

The aim of this study is to explore the integration of digital technologies in contemporary hydropower plant operations, focusing on enhancing the efficiency, reliability, and overall performance. The objective is to investigate the impact of technologies such as artificial intelligence, smart energy systems, smart grid, DTs, and the industrial internet of things. The methodology involves a comprehensive review of these technologies and their applications, including data acquisition from various sensors, real-time data analysis, and comparisons with historical data for optimization and maintenance. This study highlights significant benefits, such as improved energy production, enhanced operational safety, reduced maintenance costs, and extended equipment lifespan. DTs offer a robust framework for simulating and optimizing plant performance under various conditions, while AI approaches improve forecasting and maintenance strategies, leading to better scheduling and reduced operational risks. Integrating these advanced digital technologies enables hydropower plants to achieve greater efficiency, flexibility, and sustainability, contributing to more stable and optimized electric grid management [57].

The development and integration of a DT model for a hydroelectric power plant, as presented in this study, represents a significant advancement in the application of DT technology within the realm of hydroelectric power generation. This innovative approach focuses on the specific challenge of error prediction and prevention for critical sensors, particularly the pressure sensor, which is situated post-inlet valve. Unlike traditional anomaly detection methods reliant solely on numerical computation and historical sensor data, the methodology proposed in this study integrates advanced DT capabilities directly into PLCs. This integration not only enables real-time synchronization between physical and virtual environments but also facilitates continuous monitoring and proactive anomaly detection. By tailoring the DT model to the unique operational dynamics of a real hydroelectric power plant in Turkey, this study provides comprehensive insights into the plant’s structural and automation systems. The systematic application of DT calculations and robust control algorithms enhances operational efficiency and offers a scalable framework to improve the reliability, efficiency, and resilience of power generation systems. Experimental validation demonstrates the practical efficacy of this paper’s approach in effectively managing sensor failures, detecting anomalies, and ensuring an uninterrupted energy supply. Moreover, embedding DT capabilities directly within PLCs enhances the system’s responsiveness to sensor-related issues, thereby minimizing downtime, preventing production losses, and contributing significantly to the advancement of DT technology in practical industrial applications. This study fills a critical gap in the literature by providing a context-specific application of DT technology tailored for automation in hydroelectric power plants, thereby paving the way for further developments and applications across diverse industrial sectors.

## 2. Methodological Approach and Infrastructure Overview

A digital twin (DT) represents a sophisticated virtual counterpart of a physical object or system, leveraging real-time data and additional sources to enhance decision-making through continuous learning and adaptive reasoning. Unlike traditional multi-sensor monitoring, a DT seamlessly integrates real-time data acquisition, advanced simulations, and predictive analytics, providing a comprehensive digital portrayal of the physical system.

DTs span various dimensions tailored to industrial applications, starting with 1D for real-time monitoring of individual components like temperature and pressure. Progressing to 2D, DTs integrate data from multiple sources, enabling comprehensive system monitoring. In 3D, they utilize spatial representations crucial for applications like building information modeling. In 4D, DTs introduce temporal dynamics for dynamic simulation and predictive analytics, which is beneficial in manufacturing. Moreover, in 5D, DTs integrate AI-driven analytics for autonomous decision-making in complex systems, enhancing operational efficiency and resilience.

The approach outlined in this paper extends into 2D DTs by integrating data from multiple sources and components, allowing for comprehensive monitoring and basic interactions between system elements. This broader perspective enhances the ability to capture interdependencies and dynamics across various operational facets. The presented model then moves into 3D DTs, incorporating detailed three-dimensional representations of physical assets to enhance the spatial context and visualization capabilities. This dimension is particularly vital in applications such as building information modeling, where accurate spatial representations facilitate efficient design, construction, and maintenance planning. Furthermore, aspects of 4D DTs are integrated by enabling time-based simulations and predictive analytics. This capability supports proactive maintenance strategies and operational optimization, enabling anticipation and mitigation of potential issues before significant impacts on system performance occur.

While this study exhibits characteristics of both 3D and 4D DTs, emphasizing real-time synchronization, advanced anomaly detection algorithms, and precise fault detection, it does not fully achieve the comprehensive predictive–prescriptive analytics and autonomous control typically associated with 5D DTs. The primary focus of this study remains on enhancing operational efficiency and resilience through the integration of real-time data and dynamic modeling techniques, which are foundational to effective digital twin frameworks.

In the context of a hydroelectric power plant, this study integrates a network of strategically placed sensors, including pressure, temperature, and vibration sensors, to monitor critical components. These sensors transmit real-time data to a centralized processing unit, where data fusion techniques and communication protocols like OPC UA ensure seamless integration. The DT utilizes sophisticated mathematical and physical models to replicate the plant’s dynamics accurately. Hydraulic models simulate water flow through turbines, mechanical models capture turbine operation intricacies, and electrical models focus on generator performance. These models are meticulously calibrated using historical and real-time data to ensure high fidelity and precision. Additionally, the DT employs advanced machine-learning algorithms and statistical methods to predict potential failures and maintenance requirements, optimizing operational efficiency. Anomaly detection algorithms continuously analyze sensor data to identify deviations from normal operations, enabling real-time fault detection and diagnostics to ensure the reliability and safety of the hydroelectric power plant.

This section aims to elucidate the methodological approach of the study and detail its hardware and software infrastructure. Subsequent sections provide a concise explanation of the DT technology and comprehensive descriptions of the structural and automation systems of the selected hydroelectric power plant case study.

### 2.1. Digital Twin Application

The digital twin model developed in this study for the hydroelectric power plant is characterized by three primary dimensions: physical, operational, and functional. These dimensions collectively enable a comprehensive simulation and analysis of the plant’s performance.

Physical Dimension: The DT model accurately replicates the physical structure and components of the hydroelectric power plant. This includes detailed modeling of the hardware, mechanical systems, and equipment such as sensors, turbines, and generators. By mirroring the physical aspects, the model provides a virtual representation that aligns closely with the actual infrastructure of the plant.

Operational Dimension: This dimension focuses on the real-time operational data and processes of the plant. It incorporates data from various sensors measuring parameters like the water levels, pressure, flow rate, and temperature. The operational dimension ensures that the DT can monitor and analyze the plant’s performance in real time, facilitating an immediate response to any operational changes or anomalies.

Functional Dimension: The functional dimension encompasses the operational functionalities and processes of the hydroelectric power plant. It includes the simulation of energy production processes, the operational principles of turbines and generators, and overall performance metrics. This dimension is critical for optimizing the plant’s efficiency and identifying areas for performance improvement.

**Figure 1:**
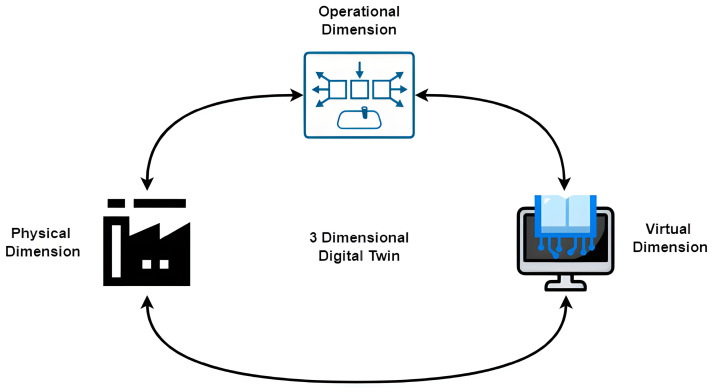
Basic structure of the digital twin model used in this study.

Figure 1 provides an illustrative depiction of a typical DT model. Notably, the architecture of DTs establishes a seamless connection between the real and virtual systems, facilitating the detection of situational changes and anomalies therein.

This system, which creates a virtual replica of a hydroelectric power plant linked to the physical plant, enabling simulations and anomaly detections, qualifies as a DT. It embodies the core features of a DT, such as real-time data integration, high-fidelity modeling, predictive analytics, and simulation capabilities. In contrast, industrial automation systems (ICSs) are more focused on real-time control and process automation, highlighting significant differences in the objectives, data usage, visualization, and interactivity. Although it works with real-time data concerning the developed system, it uses a part of a DT system as its infrastructure. Estimation regarding data usage is not recommended in this study. In Section 6, suggestions regarding data use and prediction studies are made in the section on future studies. Consequently, within the scope of this study, the capability of anomaly detection has been applied to a hydroelectric power plant. This implementation entails the utilization of various sensor data, with the example of system inlet pressure chosen to clarify the proposed model in depth. While the paper focuses on this specific case example, it is noteworthy that the same methodology can be extended to all the other components within the system.

### 2.2. Power Plant Structure

**Figure 2:**
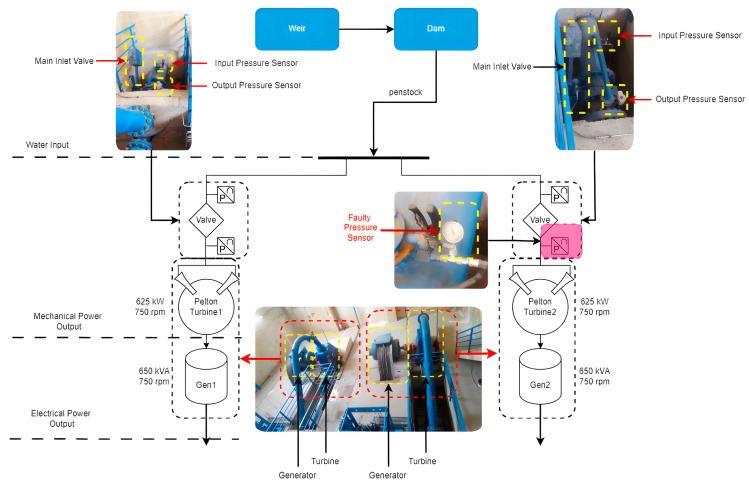
Process and instrumentation diagram of the plant.

The application of the DT technology is implemented in a real hydroelectric power plant located in Turkey. Comprising two units, each with a power output of 2 × 625 kW, this plant operates as a river-type facility, featuring a single weir and a dam. Water is conveyed approximately 3 km from the weir to the dam via a closed, unpressurized channel. Subsequently, the accumulated water in the dam is directed to the power plant through a penstock, where it is separated upon entry. Governed by valves situated at each turbine inlet, the water is then channeled to the turbine wheels via nozzles. The mechanical power thus generated is converted into electrical power by synchronous generators linked to the turbine shaft. A process and instrumentation diagram explaining the operational configuration of the power plant is provided in Figure 2.

**Figure 3:**
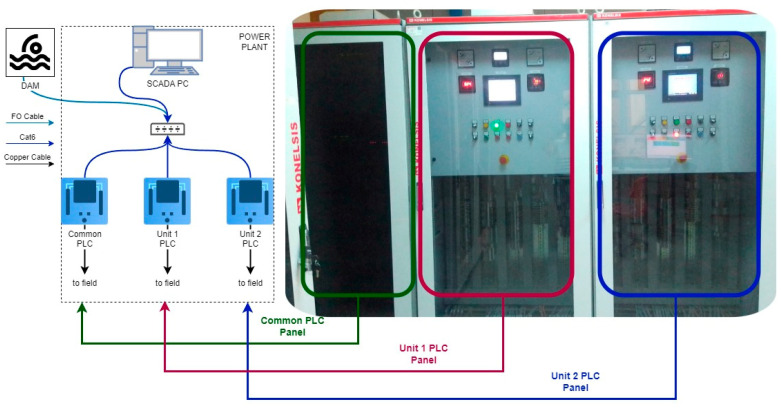
Automation topology of the plant, showing the network of PLCs and SCADA system used for monitoring and controlling the operations.

The facility’s automation system is presently managed through PLCs. Leveraging both digital and analogue channels, the PLCs effectively capture data from all the sensors and contacts within the switchboard. Moreover, they facilitate the acquisition of data through communication protocols like Modbus TCP/IP. Figure 3 illustrates the automation topology of the plant, providing a visual representation of its operational framework.

### 2.3. Automation Infrastructure

All the data generated within the power plant is aggregated within the PLCs and subsequently relayed to the user interface via the supervisory control and data acquisition (SCADA) system. Simultaneously, commands initiated by the user are transmitted back to the PLCs. Data acquired by each PLC comprises both digital and analog components. Analog data, derived from physical measurements captured by field sensors, are represented in various formats, such as 4–20 mA or 0–10 V, corresponding to the analog inputs of the PLCs. Subsequently, these values undergo scaling within the PLC, facilitating the derivation of the real physical quantities. These quantifications play a pivotal role in ensuring the robust operation of the power plant. For instance, in the event of an increase in the temperature readings from the windings or bearings of the generator, protective measures are activated to safeguard the unit. While these protective mechanisms are essential for maintaining the security of the power plant, they may occasionally result in production losses due to operational errors.

**Figure 4:**
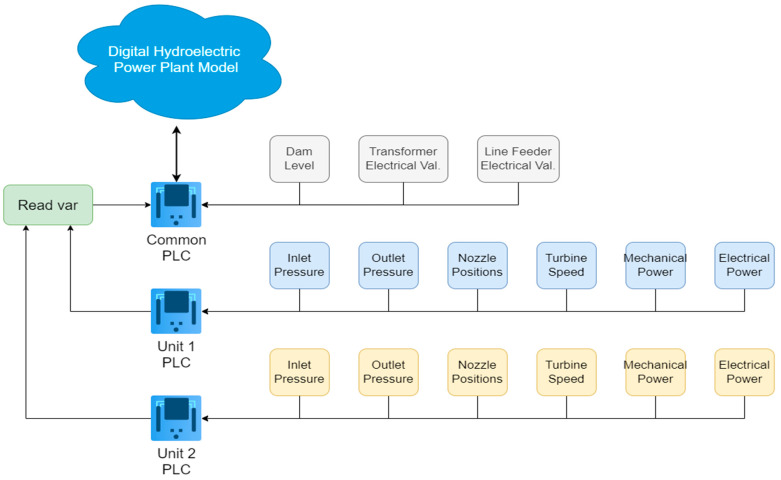
Automation infrastructure of the power plant.

The DT model of the power plant is constructed within a common PLC platform. Data pertaining to individual units are retrieved via the Modbus TCP/IP protocol. Mathematical models are then formulated based on the acquired data, establishing the interrelationships between various parameters. Figure 4 offers a graphical depiction of the data retrieval mechanism from both the common PLC and individual units, ultimately leading to the generation of the DT model. Analog values encompassing parameters such as the pressure, speed, power, and current, acquired by the units, are transmitted to the common PLC through communication channels. Additionally, the common PLC reads electrical data within its system. Subsequently, leveraging the data collected within the common PLC, a DT model of the power plant is constructed.

## 3. Development and Implementation of the Digital Twin Model

**Figure 5:**
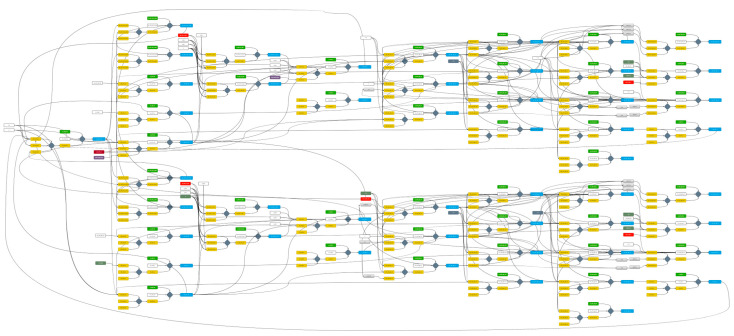
Comprehensive DT model of the plant, showing the integration of the sub-models and their interrelationships.

In this study, a comprehensive DT model of a real hydroelectric power plant located in Turkey has been developed, as depicted in Figure 5. This model is a combination of separate DTs for each piece of equipment that constitutes the plant. As shown in the figure, the full DT model of the plant is highly detailed and complex. Therefore, it was not feasible to explain the entire model in detail within the scope of this paper. Instead, one of the sub-digital twin models that constitute the main model has been selected and explained in detail to effectively demonstrate the functionality of the developed DT. This procedure, presented as an example, was repeated using a similar method for all the other equipment in the plant to create all the sub-digital twins. Subsequently, the main DT model shown in Figure 5 was developed by combining the sub-digital twins, taking into account their interrelationships.

The sub-digital twin model for the water pressure value, referred to as the outlet pressure and located after the inlet valve of Unit 1, was chosen as a case sample to clarify the DT infrastructure. This variable was selected because a fault in the power plant, caused by this sensor, resulted in a shutdown. Using the developed DT model, the correct sensor value was generated, allowing the plant to resume production. This demonstrated the DT’s importance and functionality during a real event.

The adaptation of the DT to the automation system initially presented a significant challenge, as the system relies on processing and controlling the values obtained from the sensors. Implementing the DT within the PLC necessitated a comprehensive overhaul of the entire automation system architecture. The integration of the DT with the PLC involved mapping each sensor input to corresponding variables within the DT model. This was achieved by developing custom blocks within the PLC programming environment, ensuring that real-time data from the sensors could be accurately mirrored and processed within the DT. Integration with the SCADA system required establishing communication protocols such as OPC UA and Modbus TCP/IP to facilitate seamless data exchange. This allowed the SCADA system to access both the real-time sensor data and the predictive outputs from the DT, enabling comprehensive monitoring and control.

In this study, three distinct digital twin parameters—dt0, dt1, and dt2—are employed to address the inherent complexity and variability within the hydroelectric power plant’s sensor data. The use of multiple parameters enhances the data reliability and accuracy in a system where sensor failures and communication issues can occur simultaneously or at different times. Each parameter leverages different algorithms and data sources, capturing various aspects of the data to mitigate the limitations of individual sensors and communication channels. For example, dt0 might be optimized for scenarios requiring high temporal resolution, while dt2 could be more robust against outliers or noise. This multi-faceted approach allows for comprehensive validation of the sensor data, improving the system’s resilience against faults and ensuring data accuracy. Consequently, this strategy not only enhances the reliability of the digital twin model but also supports more precise monitoring and control, which are crucial for the efficient operation of the hydroelectric power plant. While not all the sensor data may support multiple validation methods due to technical or practical constraints, increasing the validation points for the available data sources remains a viable strategy. This contributes significantly to improving the overall data quality and reliability, which are essential for accurate functioning and decision-making in complex systems. The appropriate ‘dt’ calculation method is selected among dt0, dt1, and dt2 either automatically by the control algorithm, as described in Section 3.2, or manually by the operator based on system requirements and conditions.

**Figure 6:**
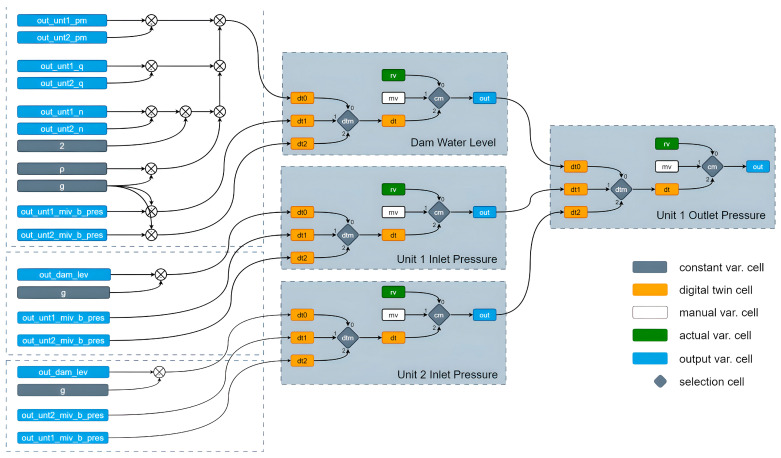
Data flow diagram for the DT model focusing on the outlet pressure sensor.

Figure 6 shows the process used to obtain the DT value of the outlet pressure. In this figure, the output of the last block labelled ‘Unit 1 Outlet Pressure’ provides the value of the outlet pressure. Three options are used to derive this value: ‘rv’ indicates the real value measured by the pressure sensor, ‘mv’ indicates a manual value that can be set by the operator to manually determine the outlet pressure, typically used for testing and observing system responses to various operating conditions, and ‘dt’ indicates the value calculated by the DT block. 

The diagram shown in Figure 6 provides a detailed view of how the digital twin model monitors and controls the pressure sensor values after the main inlet valve in a hydroelectric power plant. The digital twin cells (dt0, dt1, dt2) calculate the pressure values using different methods and select the most accurate value for system monitoring and control. This process helps in detecting anomalies and taking necessary actions to ensure the efficient and accurate operation of the system.

The components and connections within this system include constant variable cells, digital twin cells, manual variable cells, actual variable cells, output variable cells, and selection cells. The color codes used in the diagram are as follows: gray for constant variable cells, orange for digital twin cells, white for manual variable cells, green for actual variable cells, blue for output variable cells, and gray diamonds for selection cells. 

The constant variable cells consist of ρ for fluid density and g for gravitational acceleration, which are used in hydrostatic pressure calculations. 

The digital twin cells (dt0, dt1, dt2) use different algorithms and data sources, as previously detailed, to calculate the pressure value, thereby enhancing the accuracy of the sensor data and facilitating anomaly detection.

Manual variable cells (mv) allow the operator to manually enter values, as used for testing and observing system behavior under various conditions. 

Actual variable cells (rv) represent the current state of the system as they obtain actual values from the sensor.

The output variable cells (out) provide the resulting pressure value used for system monitoring and control.

Selection cells, namely dtm and cm, determine which digital twin method (dt0, dt1, dt2) is used and which value (dt, mv, rv) is selected as the output, respectively.

The data flow and calculations involve multiple variables and steps. For the dam water level, the input values include out_dam_lev (dam water level) and g (gravitational acceleration). These are used to calculate the dam water level using three different digital twin methods (dt0, dt1, dt2). The selection cell (dtm) selects the appropriate digital twin method, and the output cell (cm) selects the output value from rv, mv, or dt. The final selected dam water level is sent to the out cell.

For the Unit 1 and Unit 2 inlet pressure, the input values are out_unt1_miv_b_pres and out_unt2_miv_b_pres (pressure values before the main inlet valve for Units 1 and 2), ρ (fluid density), and g (gravitational acceleration). These values are used to calculate the inlet pressure using the three different digital twin methods (dt0, dt1, dt2). The selection cell (dtm) selects the appropriate method, and the output cell (cm) chooses the output value from rv, mv, or dt. The final selected inlet pressure value is sent to the out cell.

For the Unit 1 outlet pressure, the input values include out_dam_lev (dam water level), g (gravitational acceleration), and out_unt1_miv_b_pres and out_unt2_miv_b_pres (pressure values before the main inlet valve for Units 1 and 2). These are used to calculate the outlet pressure using the three digital twin methods (dt0, dt1, dt2). The selection cell (dtm) determines the method, and the output cell (cm) selects the output value from rv, mv, or dt, with the final selected outlet pressure value sent to the out cell.

### 3.1. Mathematical Framework for Calculating ‘dt’ Values

As mentioned in the previous section, the ‘dt’ value is calculated using several techniques, designated as dt0, dt1, and dt2, each employing a different method. The mathematical background to these methods is provided by the procedures outlined in Equations (1a)–(1c), which serve as the foundation for the selected example of the DT model of a pressure value.

unt1_miv_a_pres=(1a)dam_lev10,197(1b)unt1_miv_b_pres(1c)unt2_miv_b_pres

where

unt1_miv_a_pres: water pressure after Unit 1 inlet valve (bar)
dam_lev: dam water level (m)
unt1_miv_b_pres: water pressure before the Unit 1 inlet valve (bar)
unt2_miv_b_pres: water pressure before the Unit 2 inlet valve (bar).

Equation (1a), as the first method used to calculate the pressure value, utilizes data from the loading pool level. The pressure value at that point is calculated by dividing the loading pool level by the 10,197 from Equation (4).

The hydrostatic pressure equation, described by Equation (2), plays a fundamental role in fluid mechanics [58]. It illustrates how the pressure *P_pres_* at a depth *H* in a fluid column is determined by the fluid’s density ρ, gravitational acceleration g, and height of the fluid column *H*:

(2) $$H=\frac{P_{pres}}{g\times \rho }$$

where

*H*: height (m)
g: gravitational acceleration (m/s^2^)
*P_pres_*: pressure (Pa)
*ρ*: fluid density (kg/m^3^).

Equation (2) is derived from hydrostatic principles, which are crucial in various applications, such as ocean depth calculations, barometer design, and fluid behavior analysis. The pressure can be converted from bars to Pascals by multiplying by 10^5^. Thus, Equation (3) restates the hydrostatic pressure equation in units of bars:

(3) $$H=\frac{P_{bar}\times 10^{5}}{g\times \rho }$$

For standard gravitational acceleration g=9.81  m/s^2^ and fluid density ρ=1000 kg/m^3^, Equation (4) simplifies the height calculation.

(4) $$H\approx P_{bar}\times 10,197$$

In Equation (1b), the pressure sensor value on the same line before the Unit 1 inlet valve is taken as a reference. Equation (1c) describes the division of the penstock pipe from the loading dock to the power plant into two sections connected to Units 1 and 2, ensuring pressure equality.

The calculation methods used to obtain the dam water level are shown in Equations (5a)–(5c). These values are derived from the calculation of the loading pool level using three different mathematical methods, similar to Equations (1a)–(1c). These methods can be employed to verify the obtained values using different approaches.

dam_lev=(5a)Pu1 + Pu2 Qu1 + Qu2 × ρ × g × ηu1 + ηu22(5b)g×unt1_miv_b_pres(5c)g×unt2_miv_b_pres

where

*P_u_*_1_: Unit 1 mechanical power (W)
*P_u_*_2_: Unit 2 mechanical power (W)
$Q_{u1}$: Unit 1 flow rate (m^3^/s)
$Q_{u2}$: Unit 2 flow rate (m^3^/s)
*ρ*: fluid density (kg/m^3^)
g: gravitational acceleration (m/s^2^)
*η_u_*_1_: Unit 1 efficiency
*η_u_*_2_: Unit 2 efficiency
*unt*1*_miv_b_pres*: Unit 1 pre-inlet valve pressure (bar)
*unt*2*_miv_b_pres*: Unit 2 pre-inlet valve pressure (bar).

The method used for the forebay level in Equation (5a) is carried out by taking the equation shown in Equation (6) as a reference forebay. Since there are two different units here, values such as the mechanical power, flow rate and efficiency are obtained by adding the values of the two units. The mechanical power output in hydroelectric systems can be calculated using Equation (6). This equation integrates several critical factors: the fluid’s density (ρ), gravitational acceleration (g), height (*H*) of the water column, flow rate (Q), and system efficiency (*η*). This relationship highlights how the potential energy of water, influenced by its height and flow rate, is converted into mechanical energy through turbines. The efficiency term (*η*) accounts for the losses in the system, providing a realistic measure of the power output. This equation is pivotal in hydroelectric power generation, enabling the design and optimization of turbines and the overall system performance [57,58].

(6) $$P_{m}=\rho \times g\times H\times Q\times \eta$$

where

*P_m_*: mechanical power (W)
*ρ*: fluid density (kg/m^3^)
g: gravitational acceleration (m/s^2^)
*H*: height (m)
Q: flow rate (m^3^/s)
*η*: efficiency.

Equations (5b) and (5c) convert the pressure values into altitude values as per Equation (4), and the mechanical power equation is derived from Equation (6).

### 3.2. Control Algorithms

This section describes the control algorithms used to detect anomalies in water pressure values through a DT model. Before delving into a detailed explanation, Table 5 provides the parameters used in the control structure throughout this section.

**Figure 7:**
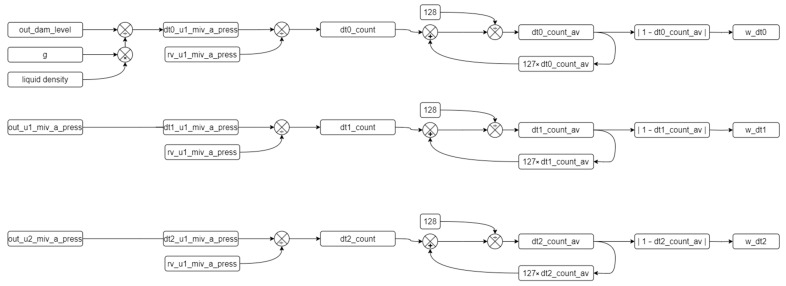
Data flow diagram of the DT.

Three different approaches, designated as dt0, dt1, and dt2, can be used to calculate the pressure value using a DT. The flow diagram illustrating how the dt0, dt1, and dt2 values are obtained, as well as the filtering software diagram, is shown in Figure 7. The algorithm of this diagram is defined in Algorithm 1.
**Algorithm 1.** Digital twin data creation algorithm.Procedure defining dt values    While (true)    dt0_u1_miv_a_press ← out_dam_level × *g* × liquid_density    dt1_u1_miv_a_press ← out_u1_miv_a_press    dt2_u1_miv_a_press ← out_u2_miv_a_press    end while

The calculation results from the dt inputs are transferred to variables. Three inputs are derived based on the reference variable from Equation (1), and three corresponding dt variables are obtained. The average of each dt variable is calculated, and a filtering process is applied to reduce the impact of instantaneous changes. The algorithm for this process is defined in Algorithm 2.
**Algorithm 2.** Algorithm for the filtering and smoothing values.Procedure filtering and smoothing values    while (true)     for n = 0; n ≤ 2; n++       dt(n)_count = dt(n)_u1_miv_a_press/rv_u1_miv_a_press       dt(n)_count_av = (dt(n)_count + (127 × dt(n)_count_av))/128     end for    end while

The selection of the number 128 as a 7-bit variable represents a deliberate choice within statistical analysis, wherein the magnitude of the change in the incoming values directly influences its contribution to the dataset’s average. By increasing this designated number, the proportional impact of new incoming values on the dataset’s average diminishes, specifically by a factor of 1/128th of the total. This strategic decision serves to mitigate fluctuations within the dataset by assigning less weight to recent values, thereby enhancing the dataset’s stability and reducing the undue influence of transient data points on analytical outcomes. Additionally, the per unit value of the variable is obtained for ease of operation. The difference between the per unit value and the reference value of 1 is calculated and used to determine the error rate. This determination is defined in Algorithm 3.
**Algorithm 3.** Algorithm for the absolute values.Procedure defining absolute values.    while (true)     for n = 0; n ≤ 2; n++       w_dt(n) = | 1 − dt(n)_count_av |     end for    end while

**Figure 8:**
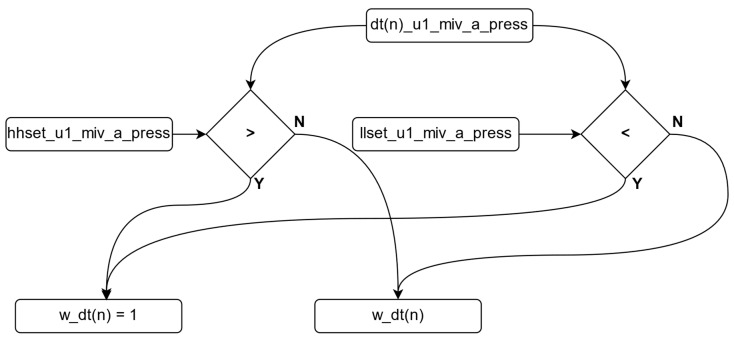
Checking algorithm for the DT variables.

As a result of this process, the dt variable with the value closest to 0 is identified as the variable most closely approximating the real value. The dt values are continuously re-calculated to ensure they remain within the specified ranges, which are determined by the upper and lower limit values set by the manufacturer based on the power plant’s operating conditions. Figure 8 illustrates the algorithm flow that checks whether the dt variables fall within these reference ranges. According to the algorithm for controlling the dt variables shown in the figure, if there is no abnormal variability in the system, the calculated error value is used. However, in cases where the values fall outside the specified ranges, the weight value corresponding to that dt is set to 1, ensuring that it is not considered in the subsequent decision-making algorithm. The algorithm of this sequence is shown in Algorithm 4.
**Algorithm 4.** Control algorithm for the dt variables.Procedure controlling dt variables.    while (true)     for n = 0; n ≤ 2; n++       if dt(n) < lower_limit or dt(n) > upper_limit then           w_dt(n) = 1       else           w_dt(n) = calculated_error_value       end if     end for    end while

**Figure 9:**
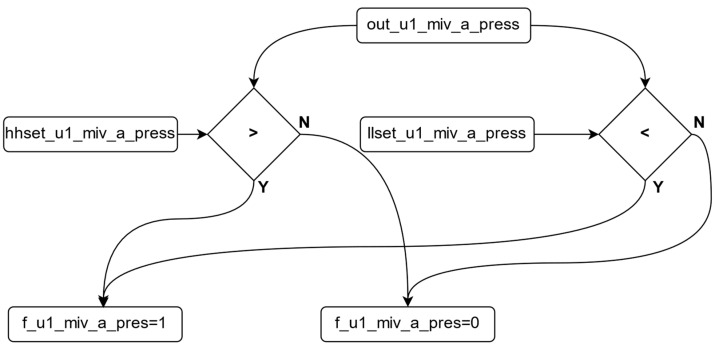
Checking algorithm for the out variable.

**Figure 10:**
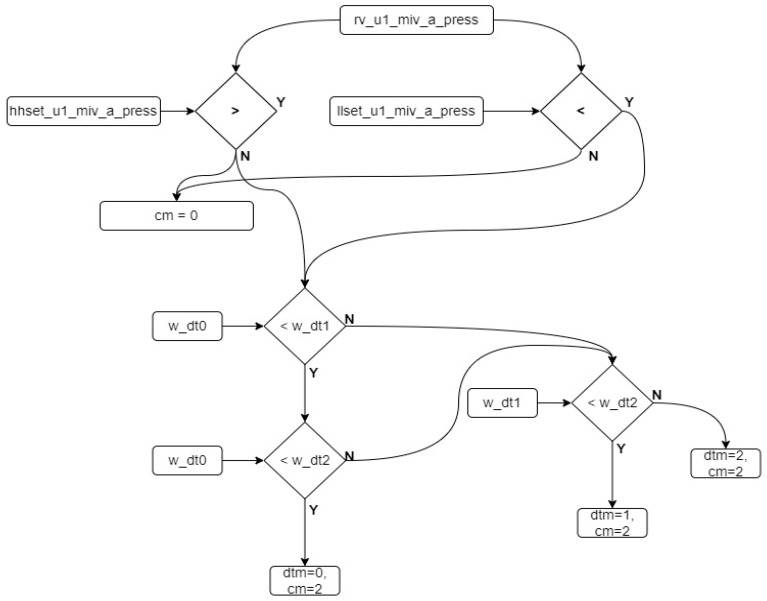
Diagram of the control and selection algorithm.

The control applied to the dt variables is similarly applied to the output variable. If a malfunction is detected in the output variable, the system identifies it as a real malfunction and generates an error to stop the system. Figure 9 illustrates the control algorithm for the output variable. After checking the output variable, if the value is outside the reference ranges, the fault variable is activated, allowing the plant to stop under normal operating conditions. The algorithm for this sequence is shown in Algorithm 5. As a result of these developed control algorithms, the system continuously monitors the variable from the sensor. If the sensor data fall within the reference values, the sensor variable is transferred to the output variable. If the sensor variable falls outside the reference values, the developed algorithm is triggered to verify the fault in the system. Figure 10 illustrates the control and selection algorithm diagram. The pseudocode for this sequence is shown in Algorithm 6.
**Algorithm 5.** Algorithm for the checking out value.**Procedure** checking out value    **while (true)**     **if** out_u1_miv_a_press > hhset_u1_miv_a_press **or**        out_u1_miv_a_press < llset_u1_miv_a_press        f_u1_miv_a_press = 1     **else**        f_u1_miv_a_press = 0     **end if**    **end while**
**Algorithm 6.** Algorithm for the checking sensor values.**Procedure** checking sensor values.    **while (true)**     **if** rv_u1_miv_a_press > hhset_u1_miv_a_press **or**          rv_u1_miv_a_press < llset_u1_miv_a_press          **if** w_dt0 < w_dt1              **if** w_dt0 < w_dt2                  cm = 2                  dtm = 0              **else if** w_dt2 < w_dt1                  cm = 2                  dtm = 2              **end if**          **else if** w_dt1 < w_dt2              cm = 2              dtm = 1          **end if**     **else**           cm = 0           dtm = 0     **end if**    **end while**

As shown in Figure 10, if the variable exceeds the reference value, the dt variables are checked. Based on this control, the dt value with the least error is transferred to the output. To transfer the dt value to the output, it is necessary to intervene in the cm and dtm variables.

### 3.3. Petri Net Model for the Fault Detection and Decision-Making Algorithm

**Figure 11:**
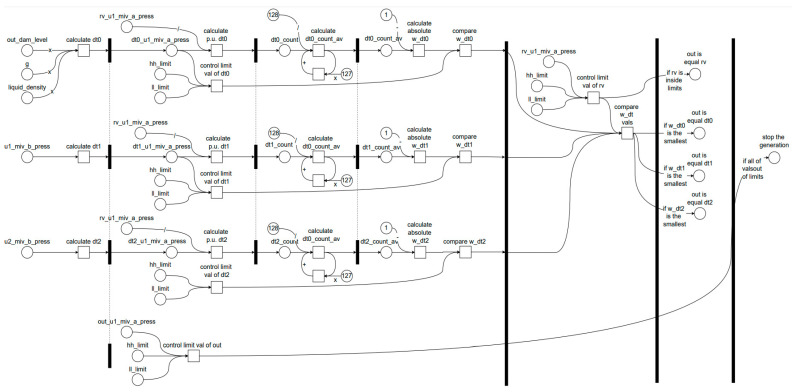
Petri Net diagram of the selection algorithm.

In this study, the Petri Net model is employed to represent the fault detection and decision-making algorithm within the hydroelectric power plant’s DT system. Figure 11 illustrates a sophisticated control logic system designed for monitoring and regulating hydraulic pressure within the power plant. This model provides a structured and formal method for capturing decision-making processes and control flows. The diagram comprises places (e.g., out_dam_level, liquid_density, rv_u1_miv_a_press) and transitions (e.g., calculate dt0, calculate dt1, calculate dt2). Tokens flow through these elements to simulate the system’s behavior. Tokens in places like out_dam_level and liquid_density represent the initial conditions. Transitions like calculate dt0 simulate the computation of dynamic values based on these conditions.

By offering a visual representation of the entire fault detection and management process, the Petri Net model maps the sequence from reading sensor values to making decisions and potentially stopping the system if necessary. The parallel structure of the Petri Net allows multiple calculations (dt0, dt1, dt2) to occur simultaneously, reflecting the concurrent nature of the system processes. Additionally, the model enables the simulation of various scenarios, including normal operations and fault conditions, allowing for thorough analysis and optimization. This model includes places and transitions that monitor sensor values and compare them against predefined limits to detect faults. Transitions such as compare dt values evaluate the calculated figures to determine the most reliable output. If a fault is detected (e.g., all the dt values are out of the limits), the transition triggers a system shutdown, ensuring the system stops to prevent damage.

The system ensures the safe and efficient operation of various components by maintaining the pressures within predefined limits and adjusting the outputs accordingly. The primary variables in this system include the dam liquid density, dam level, and hydraulic pressures at multiple points. The control logic diagram begins with measuring the dam’s liquid density and level, which are crucial inputs for calculating dt0, an intermediate variable indicating a specific pressure-related parameter. Similar calculations are carried out for dt1 and dt2, derived from distinct pressure readings, specifically rv_u1_miv_b_press.

Each calculated pressure—dt0, dt1, and dt2—is then subjected to threshold checks against the high (hh_limit) and low (ll_limit) control limits to ensure that the pressures remain within the predefined safe operating range. Control limit validation is critical to prevent potential overpressure or underpressure conditions that could compromise the system stability and safety. The diagram also incorporates averaging operations for dt0, dt1, and dt2 (denoted as dt0_count_av, dt1_count_av, and dt2_count_av) to smooth out the readings and provide a more stable control response. These averaged counts are compared against the initially calculated pressures to determine if they fall within acceptable ranges, ensuring measurement stability.

The output determination is based on selecting the appropriate output with the smallest absolute difference between the measured and average pressures (w_dt0, w_dt1, w_dt2). The final outputs (out) are confirmed by ensuring that the selected pressures are within the established control limits. If any control values exceed the thresholds, the system triggers safety mechanisms to halt power generation, protecting the overall system from potential damage. The flow of the control logic starts with the initial calculation and validation of the input pressures against the high and low limits. The intermediate values (dt0_count_av, dt1_count_av, dt2_count_av) are computed and compared with the initial pressure calculations to ensure the accuracy and stability. Based on these comparisons, the appropriate output (out) is selected from dt0, dt1, or dt2. The system continuously monitors these outputs to ensure they remain within the specified limits, highlighting the dynamic and real-time nature of the monitoring process.

In terms of safety and generation control, the system is designed to halt power generation if all the outputs fall outside the acceptable limits. This feature ensures operational safety by preventing the system from operating under unsafe conditions. Overall, this control logic diagram exemplifies the complexity and precision required in hydroelectric power plant operations, emphasizing the importance of real-time monitoring and response mechanisms to maintain system stability and efficiency.

## 4. Experimental Results

As previously discussed, a DT model and anomaly detection application were developed for a real hydroelectric power plant. Due to the complexity of the full DT model, a sub-digital twin model focusing on the outlet pressure sensor in Unit 1 was selected as a case study to demonstrate the DT system’s functionality. In the power plant, the pressure values are bounded by a lower limit of 24 bar and an upper limit of 28 bar, with values outside this range considered abnormal. An interface within the PLC monitors the system changes, displaying a table for the pressure values that are read and calculated in real time by the PLC, showing the calculation results and control outcomes.

**Figure 12:**
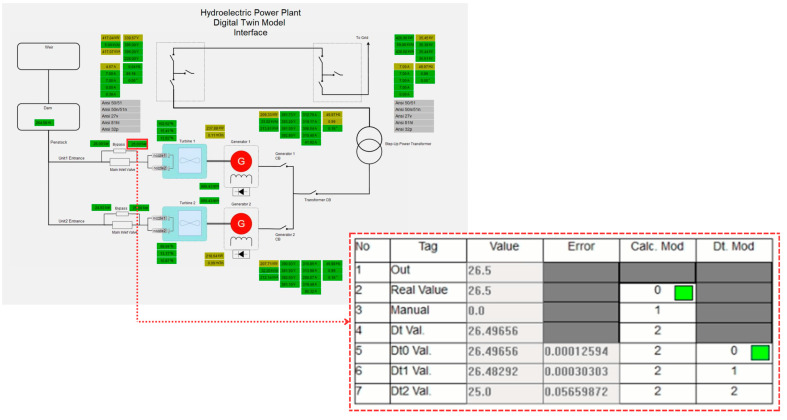
Normal operation of the system.

The algorithm was tested in three stages. In the first stage, it demonstrated that the sensor values were close to the normal value and the dt0. Figure 12 illustrates the pressure values corresponding to normal operation as displayed on the PLC’s online monitoring screen. The sensor value is read as 26.5 bars, which is within the acceptable range and very close to the dt0 value produced by the DT model, indicating normal operation. The real sensor data are used as the output, and the ‘Calc. Mod’ column in the table shows which data are currently being transferred to the output. In Figure 12, a green box in the cell where the ‘Calc. Mod’ column and the ‘Real Value’ row intersect indicates that the real sensor value is being transferred to the output. If the DT value is used instead, the green box will appear in the corresponding cell.

**Figure 13:**
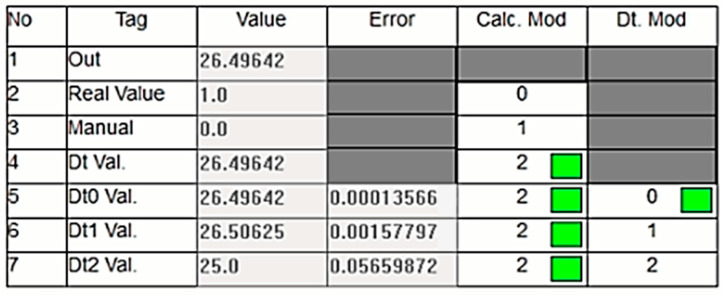
Dt0 is selected as the output due to sensor inaccuracy.

**Figure 14:**
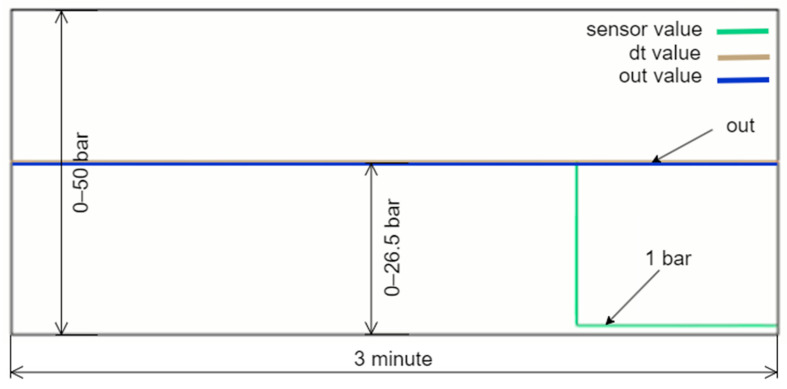
Real-time changes in the sensor and DT0 values when the sensor drops to 1 bar.

Figure 13 illustrates an experimental study conducted under a different operational condition where the sensor data decrease to 1 bar. In this case, as clearly shown in the figure, the green box indicates that the digital twin value (Dt Val.) is selected as the output since the real value measured by the sensor is considered inaccurate. As mentioned in previous sections, the DT model utilizes three different calculation options called dt0, dt1, and dt2, each employing a different technique. In the scenario depicted in Figure 13, the lowest error value is associated with dt0, so dt0 is selected as the digital twin’s output rather than dt1 or dt2. The green box appearing within the relevant cell under the Dt. Mod column highlights this situation. In this scenario, the system has seamlessly transitioned to output the Dt0 value, ensuring the continuation of the energy supply without causing any oscillation at the system’s output. Figure 14, taken from the PLC online real-time monitoring screen, illustrates the changes in the sensor value and Dt0 value at the moment when the sensor value decreases to one. The data presented here correspond to the values shown in Figure 13.

**Figure 15:**
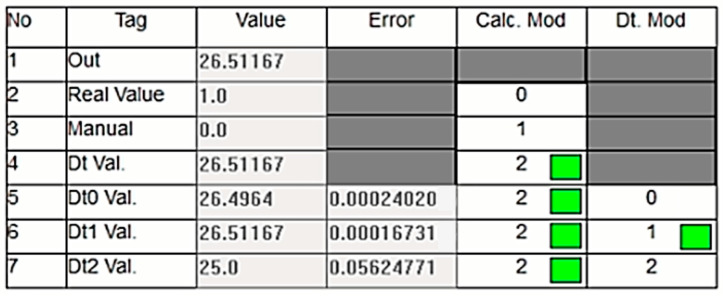
Dynamic selection of Dt1 as the output due to its smallest error.

In the system, the dt0, dt1, and dt2 values are dynamically calculated in real time. The developed control algorithm transfers the value with the smallest error to the output during this dynamic process. Figure 15 exemplifies this situation. As shown in the figure, the DT data are used because the actual sensor data are incorrect. In this instance, the dt1 data, which has the lowest error at this moment, are selected as the DT’s output.

**Figure 16:**
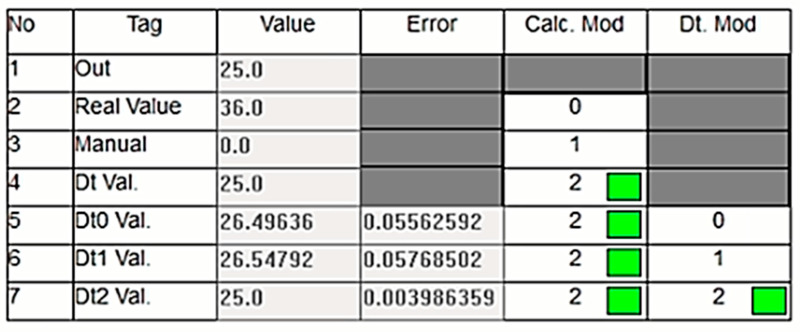
Values when Dt2 is used as the output.

**Figure 17:**
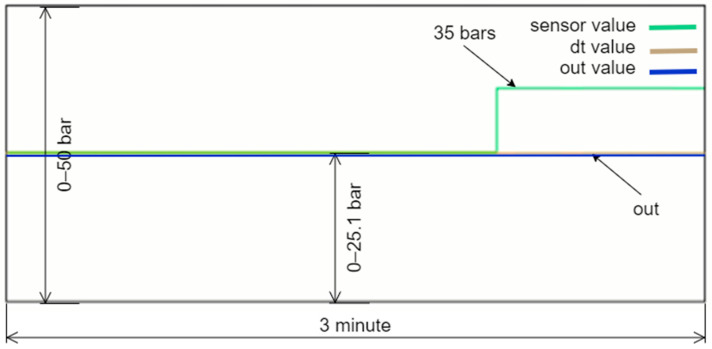
System using Dt2 as the output despite the sensor value increase.

Similar to the cases of Dt0 and Dt1, tests are conducted for the scenario where Dt2 is used as the output. Figure 16 shows the values in this scenario. As seen, the real value measured by the sensor is outside the normal limits, necessitating the use of the DT value. Among the three methods, the Dt2 value is assigned as the output of the DT since its error is closest to zero. As shown in Figure 17, despite an abnormal increase in the real sensor value, the system continues its operation smoothly by transferring the DT value to the output.

**Figure 18:**
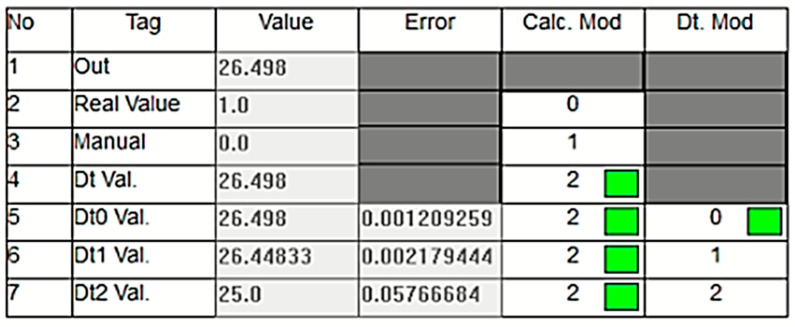
Incorrect sensor measurement (1.0 bar) causing the Dt0 output.

**Figure 19:**
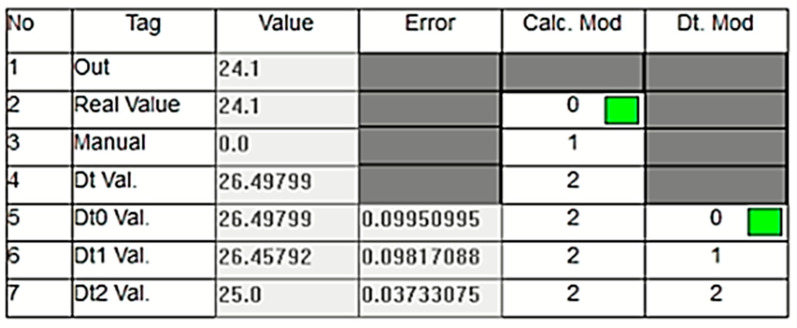
System normalizes with the ‘Calc. Mod’ as the ‘Real Value’.

Another experimental test involves transitioning from a faulty state to a normal state. This situation demonstrates that after the repair of the sensor, cable, or any other malfunction, the system detects that the error has been rectified and re-enables the transfer of sensor data to the output. Figure 18 captures the scenario where the sensor provides an incorrect measurement of 1.0 bar, resulting in the Dt0 value being transferred to the output. Once the sensor value returns to the normal ranges after the issue is resolved, the system’s operational status is restored to normal. This is depicted in Figure 19, where the ‘Calc. Mod’ is selected as the ‘Real Value’, as indicated by the green box.

**Figure 20:**
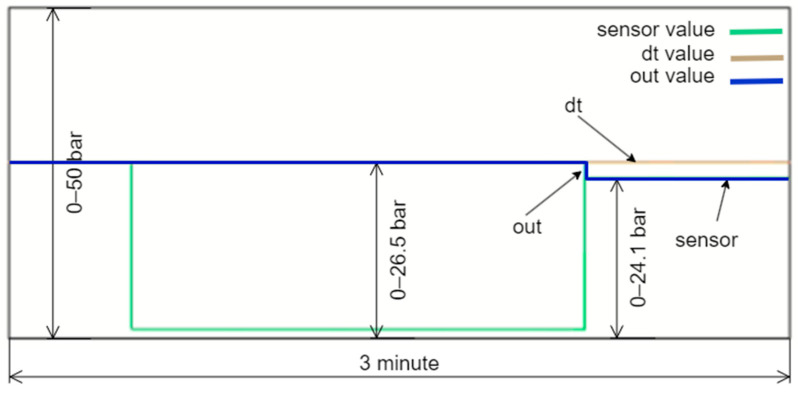
Variables during the transition to normal operation.

The state of the variables at the moment when the system transitions to normal operation is graphically represented in Figure 20. As can be seen, once the faulty sensor value returns to the normal ranges, the output value is selected as the sensor value, regardless of the Dt values.

The final experimental case conducted in this study addresses an extreme situation where a real failure occurs not only for one sensor but for the entire plant. This scenario is particularly significant because, as detailed in the previous section, the DT model relies on several parameters of the plant to calculate its output. For instance, the model utilizes data measured from other equipment to derive the actual data of a single sensor. If all the equipment in the plant is out of service, no data are received by the twin, and consequently, no output will be generated by the twin. 

**Figure 21:**
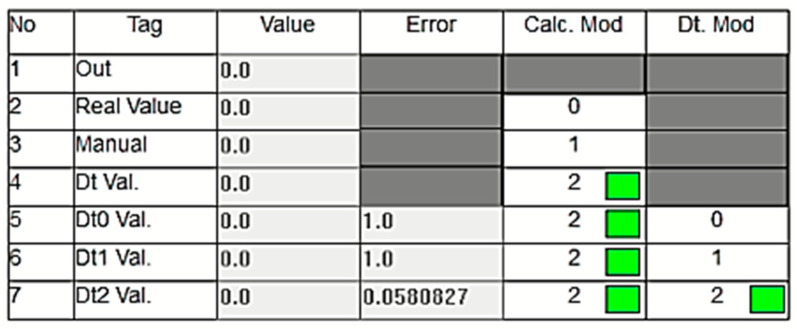
Sequential reduction of all the DT values to 0, followed by the sensor value dropping to 0.

**Figure 22:**
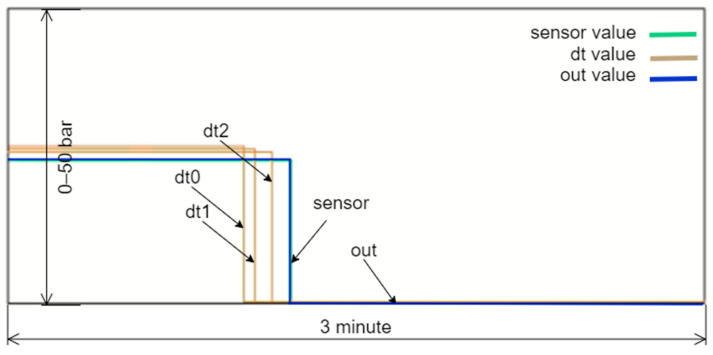
Sequential reduction of dt0, dt1, and dt2 to 0.

Figure 21 illustrates a sample situation where all the Dt values are sequentially reduced to 0, followed by the sensor value dropping to 0 as well. Figure 22 demonstrates the sequential reduction of dt0, dt1, and dt2 to 0. While checking the output value, the system eventually encounters an error when examining dt2. In the event of such a large-scale failure, the power plant will be isolated from the main grid and will remain out of service until the issue is resolved.

**Figure 23:**
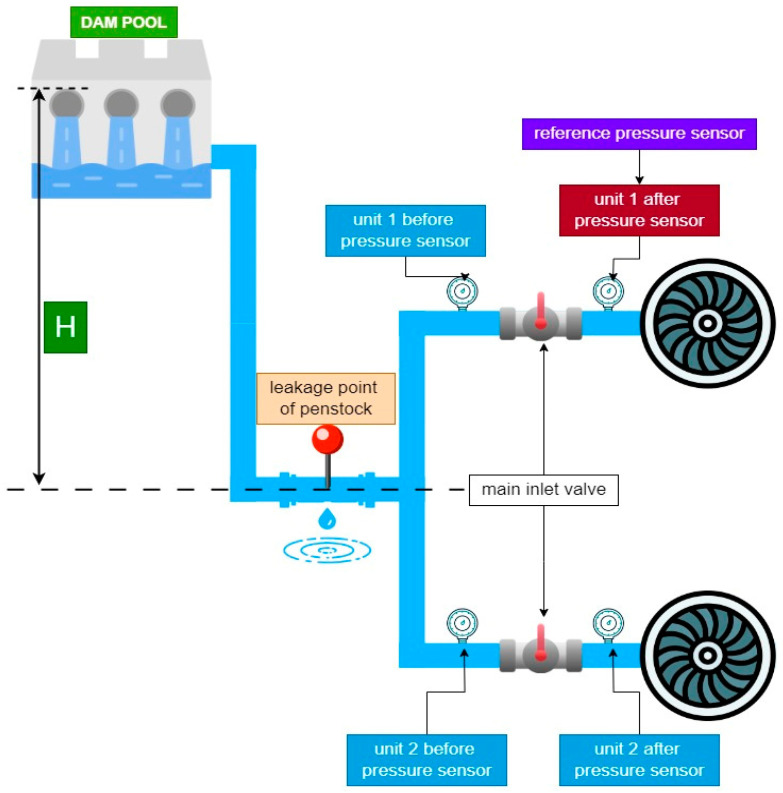
An example of a real water leakage.

A critical aspect of this study is the establishment of a malfunction judgment and analysis model capable of distinguishing between genuine system malfunctions and those arising from measurement or connection errors. This model is essential for ensuring accurate fault detection and causal analysis within the system. As depicted in Figure 23, the proposed method for detecting faults leverages changes in the state variables used as references for DTs to identify real faults.

In the event of a leak, for example, the variable ‘dam_level’ will exhibit a decreasing trend. This decrease is proportionally mirrored in other related variables, such as ‘u1_miv_b_press’ and ‘u2_miv_b_press’, due to gravitational acceleration effects. The developed algorithm continuously monitors these key variables, and when the ‘rv_u1_miv_a_press’ variable deviates from its reference values, the system begins to check other related variables to confirm the fault. The process involves several steps: monitoring key variables, comparing them against reference values, verifying faults by assessing trends across multiple variables, and triggering emergency scenarios upon confirmation. This comprehensive approach ensures that a single erroneous reading does not trigger a false alarm. The entire process occurs within a single software cycle, ensuring real-time detection and response. By leveraging the robustness of digital twin technology, which provides high-fidelity simulations of the physical system, the system can accurately differentiate between real malfunctions and those caused by transient measurement errors or connection issues. Experimental validation of the proposed approach has demonstrated its effectiveness in accurately identifying and responding to genuine malfunctions while avoiding unnecessary interventions due to measurement errors. These results underscore the reliability and efficiency of the malfunction judgment and analysis model, highlighting its significance in enhancing the operational reliability of hydroelectric power generation systems.

The experimental results presented in this section provide valuable insights into the performance and functionality of the developed digital twin model and anomaly detection application for the hydroelectric power plant. Through a series of tests and scenarios, the efficacy of the digital twin system in detecting anomalies, managing sensor failures, and ensuring the continuity of the energy supply has been demonstrated. Notably, the system seamlessly transitions between different operational states, effectively utilizing digital twin values when sensor data are inaccurate and reverting to sensor data once normal operation is restored. Moreover, these findings underscore the significance of digital twin technology in enhancing the reliability and resilience of power generation systems.

## 5. Additional Examples of Developed DT Model Results

DTs represent a transformative technology across diverse sectors, offering substantial advancements in numerous applications. For instance, in bearing fault diagnosis, researchers have developed a multi-degree-of-freedom model within a virtual environment to simulate vibration responses, enhancing diagnostic accuracy. Innovations like the frequency-domain bi-directional long short-term memory cycle generative adversarial network have further refined this approach by mapping the complex relationships between vibration responses and measured signals [59]. Addressing the sample imbalance in fault diagnosis, another study introduced a digital twin framework that synthesizes diverse fault samples using data-driven and model-based techniques, minimizing reliance on physical signals [60].

Building on these precedents, this study focuses on developing a comprehensive digital twin model tailored for a hydroelectric power plant. Previous works such as [59,60] underscored the efficacy of digital twins in generating realistic fault scenarios and improving predictive maintenance through enhanced accuracy and reliability. In line with these achievements, the research presented in this paper aims to construct a detailed digital twin that models the plant’s physical components and operational dynamics, integrating real-time data to simulate, analyze, and predict system behavior under diverse conditions.

**Figure 24:**
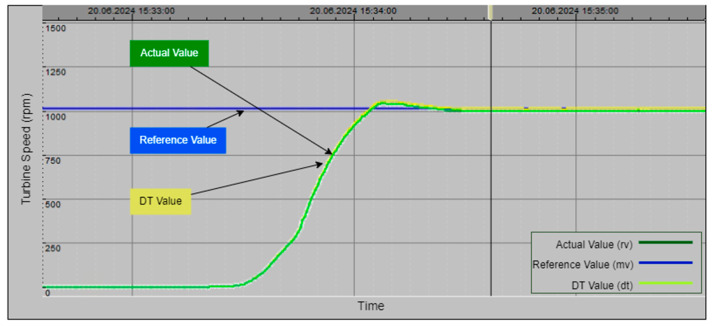
Application example of the turbine speed.

**Figure 25:**
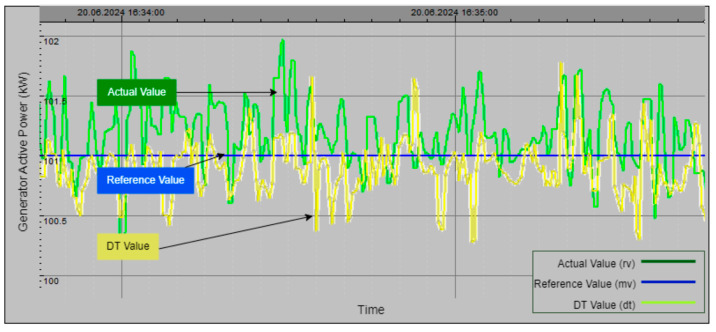
Application example of the active power.

**Figure 26:**
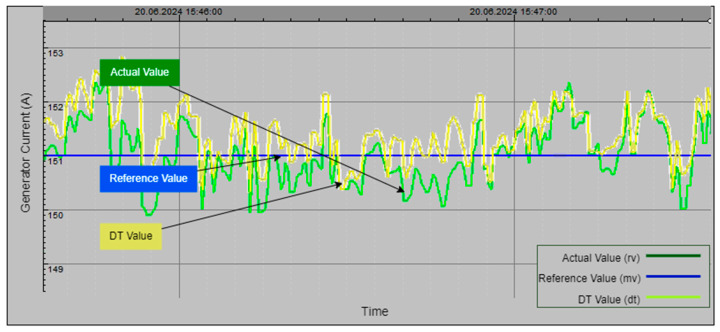
Application example of the Unit 1 generator current.

**Figure 27:**
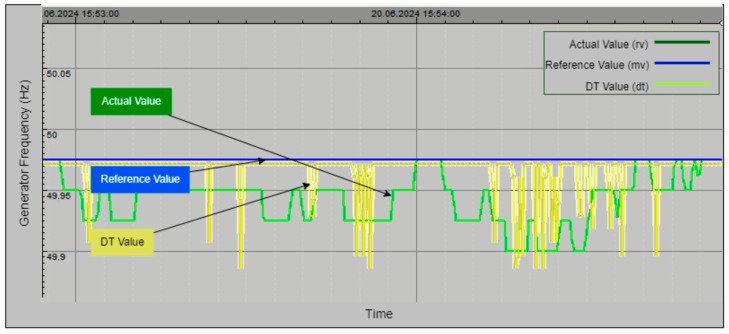
Application example of the Unit 1 frequency.

While this paper specifically details the application of the developed DT model to pressure sensors, it encompasses various aspects of the hydroelectric plant, including the water flow dynamics, turbine performance, generator efficiency, and overall plant operation. This model supports real-time monitoring and anomaly detection, enabling prompt identification and resolution of deviations from normal operation. Figure 24, Figure 25, Figure 26 and Figure 27 further illustrate additional examples of the developed DT model’s capabilities.

Figure 24 illustrates the practical application of turbine speed monitoring in the hydroelectric power plant. The graph compares three values: real-time actual speed (rv) obtained from the plant’s sensor (green line), manually set speed value (mv) for testing purposes (blue line), and speed value (dt) generated by the DT model (yellow line). Initially starting from zero, the turbine speed gradually increases, with the DT value closely tracking the actual speed over time, while the manually set speed remains constant. This comparison highlights the DT model’s effectiveness in real-time monitoring and predictive analytics for turbine speed management, demonstrating its capability to accurately replicate and predict turbine performance.

Figure 25 depicts another practical application example, focusing on the DT model of the active power generated by the plant. The graph compares the actual value of active power (green line), manually set active power value (blue line), and DT value of active power (yellow line) over time. The fluctuating nature of the actual power generation is accurately mirrored by the DT value, underscoring the model’s capability to replicate real-time performance. This alignment highlights the DT model’s effectiveness in providing accurate and reliable monitoring of active power, which is crucial for optimizing operations and ensuring efficient power generation.

Figure 26 illustrates the DT model of the Unit 1 generator current in the hydroelectric power plant. The graph compares the actual generator current (green line), manually set generator current (blue line), and DT-generated generator current (yellow line) over a specified time period. Fluctuations in the actual generator current are closely followed by the DT value, demonstrating the model’s high accuracy in simulating generator dynamics. The stable manual value provides a consistent baseline for reference, emphasizing the DT model’s effectiveness in accurately replicating real-time data and supporting optimal generator performance.

Figure 27 showcases the DT model of the Unit 1 frequency in the plant. The graph compares the actual frequency (green line), manually set frequency (blue line), and DT frequency (yellow line) over time. Variations in the actual frequency are accurately tracked by the DT model, highlighting its precision in simulating real-time frequency behavior. The stable manual frequency serves as a reference point, further validating the DT model’s ability to provide reliable and accurate modelling.

Collectively, Figure 24, Figure 25, Figure 26 and Figure 27 demonstrate the effectiveness and precision of the DT model in simulating and monitoring critical parameters within a hydroelectric power plant. The close alignment between the actual values and DT-generated values underscores the model’s capability to provide accurate monitoring, optimize plant performance, and swiftly identify anomalies for proactive maintenance. This advanced monitoring capability supports the plant’s overall goal of sustaining high levels of energy production and operational excellence.

## 6. Discussion

This study represents a significant advancement in the application of DT technology within hydroelectric power generation. By integrating DT capabilities directly into PLCs, the presented approach achieves real-time synchronization between the physical and virtual environments, facilitating continuous monitoring, proactive anomaly detection, and seamless operational transitions. This integration is crucial for enhancing the reliability, efficiency, and resilience of power generation systems.

Based on a comprehensive analysis, this study primarily aligns with the characteristics of a 3D DT, emphasizing detailed spatial representations and the integration of multiple data sources. Additionally, it incorporates elements of 4D DTs through time-based simulations and predictive maintenance strategies. A detailed assessment highlights how this study aligns with these dimensions.

This study emphasizes real-time data integration and collection from various sensors, aligning closely with the foundational principles of 3D DTs. However, it recognizes a gap in the comprehensive integration of historical and external data sources. Although advanced simulations and predictive analytics are utilized, they demonstrate the essential characteristics of both 3D and 4D DTs, yet they are not as fully developed as those found in mature DT applications. The emphasis on continuous learning and dynamic recalibration resonates well with the capabilities inherent in 3D and partially in 4D DTs.

Advanced anomaly detection algorithms and precise fault detection mechanisms are integral to this study, consistent with the functionalities expected in 3D and 4D DTs. The holistic optimization approach mirrors the principles of a general 3D digital twin, with some aspects aligning closely with 4D models. This study ensures real-time synchronization with continuous updates, a characteristic shared by both 3D and 4D DTs.

While the proactive maintenance strategies are aligned with the predictive capabilities typical of 4D DTs, they may not reach the advanced levels seen in 5D models. This study emphasizes in-depth analysis using various data sources, typical of 3D DT applications, although it does not fully encompass the broader scope of 4D or 5D models. This study employs advanced communication protocols that meet 3D DT standards, although they do not encompass the extensive protocols required for higher-dimensional models. High-resolution models and interactive visualizations are also included, which are characteristic of 3D DTs.

In addition to the comparisons discussed above, this section provides a detailed discussion encompassing the contributions and innovations, addressing the operational challenges, experimental validation, integration and real-time processing, real-time simulations, limitations, and future research directions.

Contributions and Innovations: A key contribution of this work is the development and implementation of a comprehensive digital twin model specifically tailored for a real hydroelectric power plant in Turkey. The model incorporates a network of strategically placed sensors, including pressure, temperature, and vibration sensors, to monitor critical components. These sensors transmit real-time data to a centralized processing unit, where data fusion techniques and communication protocols such as OPC UA ensure seamless and efficient integration. The digital twin employs sophisticated mathematical and physical models to accurately replicate the plant’s dynamics, including the hydraulic, mechanical, and electrical systems. These models are meticulously calibrated using real-time data to ensure high fidelity and detection.

Addressing Operational Challenges: The digital twin model developed in this study addresses several challenges commonly encountered in hydroelectric power plants. One of the primary issues is the differentiation between actual sensor faults and measurement errors. By leveraging real-time data and sophisticated modeling, the digital twin can distinguish between these scenarios, allowing for appropriate corrective actions. In cases of actual sensor failures, the system can prompt immediate maintenance actions to prevent potential damage or inefficiencies. Conversely, in cases of measurement or connection errors, the system can rely on the digital twin’s calculations to maintain operations without unnecessary interruptions. This capability not only enhances operational reliability but also reduces maintenance costs and downtime.

Experimental Validation: The experimental results presented in this study demonstrate the practical efficacy of the digital twin model and anomaly detection application in real-world scenarios. The developed system effectively manages sensor failures, detects anomalies, and ensures the continuity of the energy supply. Notably, the system transitions seamlessly between different operational states, utilizing digital twin values when sensor data are inaccurate and reverting to sensor data once normal operation is restored. This robustness and adaptability underscore the system’s potential to significantly enhance the reliability and resilience of hydroelectric power generation systems.

Integration and Real-Time Processing: The integration of digital twin capabilities directly into PLCs enhances the system’s responsiveness to sensor-related issues, further contributing to the reliability and resilience of the plant. PLCs play a critical role in the automation and control of hydroelectric power plants, and embedding digital twin functionalities within these controllers ensures real-time, accurate, and efficient processing of operational data. This direct integration eliminates communication delays that could occur if the digital twin model were housed in separate systems, thereby ensuring faster and more reliable decision-making processes.

Real-Time Simulations: Moreover, this study highlights the importance of real-time simulations in improving decision-making processes within hydroelectric power plants. The ability to conduct real-time simulations allows plant operators to anticipate potential issues and implement proactive maintenance strategies. Detection algorithms, powered by the digital twin, can detect equipment failures and operational inefficiencies, enabling timely interventions that minimize downtime and prevent production losses. These capabilities are essential for maintaining the high operational efficiency and reliability required in hydroelectric power generation.

Limitations: While this study presents a robust framework and promising results, it is essential to acknowledge the limitations and potential areas for future research. One of the limitations of the current digital twin model is its scope, which primarily focuses on specific components and sensors within the hydroelectric power plant. Expanding the model to incorporate more components and systems within the plant could provide a more comprehensive view of its operations and facilitate holistic management.

Data Storage, Data Analysis, and Data-Based Modeling: An identified area for critical improvement in this study lies in enhancing the data storage, data analysis, and data-based modeling capabilities within the digital twin (DT) framework. Effective intelligent fault diagnosis and prediction heavily rely on robust data handling and advanced analysis techniques. The current implementation presented in this study lacks comprehensive integration of historical and external data sources, which significantly hinders the realization of predictive analytics to their full potential. Future iterations will prioritize the integration of advanced data management systems and analytics tools aimed at enhancing the fault diagnosis accuracy and prediction capabilities. Moreover, the current deployment of the DT system within PLCs poses challenges in terms of data storage and management. Given that PLCs are not inherently designed for extensive data storage, effectively managing and analyzing the substantial volumes of data generated by the digital twin system can be cumbersome. Addressing this challenge involves integrating an application programming interface (API) that seamlessly connects the digital twin system to a robust database management system. Such integration will streamline the data storage, retrieval, and analysis processes, thereby enabling more precise forecasting and proactive maintenance strategies.

Future Research Directions: Ongoing efforts will focus on optimizing the control algorithms, expanding the digital twin model to encompass more plant components, and investigating digital twin applications in other industrial sectors. These advancements will not only overcome the current limitations but also pave the way for more advanced and comprehensive digital twin systems in the future.

Interoperability and Cybersecurity: An area highlighted for future improvement includes enhancing the interoperability and cybersecurity aspects within the digital twin framework. The current implementation does not fully support robust interoperability standards across diverse systems, potentially limiting the seamless integration with other industrial processes or external data sources. Moreover, while the system ensures data integrity and confidentiality through secure communication protocols like OPC UA, further enhancements are necessary to fortify the cybersecurity measures against evolving threats. Addressing these aspects will be pivotal to broadening the applicability and reliability of the digital twin model in complex industrial environments.

## 7. Conclusions

This paper presents an application of digital twin technology within the realm of hydroelectric power generation, offering a comprehensive solution for anomaly detection and operational optimization. The integration of virtual and physical environments within a unified framework, coupled with the direct embedding of digital twin capabilities into PLCs, represents a novel approach that holds immense promise for enhancing the reliability, efficiency, and resilience of power generation systems.

A key contribution of this study lies in the development and implementation of a comprehensive digital twin model tailored specifically for a real hydroelectric power plant located in Turkey. Through detailed descriptions of the plant’s structural and automation systems, including its power plant structure and automation infrastructure, the paper provides valuable insights into the operational dynamics of such facilities. The mathematical frameworks and control algorithms elucidated in the study offer a systematic approach to digital twin calculations and anomaly detection, further enhancing the utility and effectiveness of the developed model.

The experimental results demonstrate the practical efficacy of the digital twin model and anomaly detection application in real-world scenarios. By effectively managing sensor failures, detecting anomalies, and ensuring the continuity of energy supply, the developed system showcases its potential to significantly enhance the reliability and resilience of hydroelectric power generation systems. The seamless transition between different operational states, coupled with the ability to utilize digital twin values in the absence of accurate sensor data, underscores the robustness and adaptability of the developed solution.

Future research directions include a range of opportunities for further refinement and expansion of the digital twin model. Optimization of the control algorithms, integration of additional components within the power plant into the digital twin framework, and exploration of potential applications in other sectors represent promising avenues for continued advancement. Moreover, ongoing efforts to enhance the scalability, interoperability, and cybersecurity of digital twin systems will be crucial to maximizing their potential impact across diverse domains.

## Figures and Tables

**Table 1:** Software used for DTs.

Methodology	References
Aspen Plus	[35]
Thermoflow	[36]
3D Java (for Visualization)	[37]
Ansys	[38]
COMSOL	[39,40]
MATLAB	[41]
Modelica	[42]

**Table 2:** Some application examples of DTs.

System	Reference
Power plants	[36]
Wind turbines	[38]
Steam turbines	[43]

**Table 3:** Studies on energy DTs.

Main	Purpose	References
Virtual testing	Design	[35]
	Optimization	[44,45]
Process optimization	Process studies	[36,46]
	Process estimation	[39,40,47]
	Process monitoring	[43,44]
	Process training	[48]
Control		[42]
Service studies	Fault detection and diagnosis	[49]

**Table 4:** Structure of energy DTs.

Main	Purpose/System	Reference
Data-based	Cooling tower	[46]
Machine learning-based	IoT-supported	[47]
AI-based	Control and optimisation	[50]
Online analysis	Power grid	[51]

**Table 5:** Descriptions of the parameters used in the control algorithm.

Parameter	Description
out_dam_level	Output value of dam water level
gravitional_acceleration	g power (9.81)
liquid_density	Coefficient factor of liquid density (1 for water)
out_u1_miv_a_press	After main inlet valve water pressure of Unit 1
out_u2_miv_a_press	After main inlet valve water pressure of Unit 2
dt0_u1_miv_a_press	After main inlet valve water pressure digital twin 0 calculation result
dt1_u1_miv_a_press	After main inlet valve water pressure digital twin 1 calculation result
dt2_u1_miv_a_press	After main inlet valve water pressure digital twin 2 calculation result
dt(n)_u1_miv_a_press	Refers to dt0,1 and 2 values
rv_u1_miv_a_press	Sensor actual value from the PLC
dt(n)_count	Per unit (p.u.) value of dt values
dt(n)_count_av	Average of p.u. value
w_dt(n)	Weight of dt values
hhset_u1_miv_a_press	The maximum fault value of pressure value
llset_u1_miv_a_press	The minimum fault value of pressure value
f_u1_miv_a_press	Digital fault value created by the system
dtm	Digital twin selection parameter
cm	Calculation mode parameter
w_dt0	Weight of dt0 value
w_dt1	Weight of dt1 value
w_dt2	Weight of dt2 value

## Data Availability

The data presented in this study are available on request from the corresponding author. The data are not publicly available due to privacy issues.
